# Intrinsically Disordered Proteins in a Physics-Based World

**DOI:** 10.3390/ijms11125292

**Published:** 2010-12-21

**Authors:** Timothy H. Click, Debabani Ganguly, Jianhan Chen

**Affiliations:** Department of Biochemistry, Kansas State University, Manhattan, KS 66506, USA

**Keywords:** conformational selection, generalized Born, implicit solvent, induced folding, molecular dynamics, p21, p27, p53, pKID, replica exchange

## Abstract

Intrinsically disordered proteins (IDPs) are a newly recognized class of functional proteins that rely on a lack of stable structure for function. They are highly prevalent in biology, play fundamental roles, and are extensively involved in human diseases. For signaling and regulation, IDPs often fold into stable structures upon binding to specific targets. The mechanisms of these coupled binding and folding processes are of significant importance because they underlie the organization of regulatory networks that dictate various aspects of cellular decision-making. This review first discusses the challenge in detailed experimental characterization of these heterogeneous and dynamics proteins and the unique and exciting opportunity for physics-based modeling to make crucial contributions, and then summarizes key lessons from recent *de novo* simulations of the structure and interactions of several regulatory IDPs.

## 1. Introduction

The prevalence and fundamental roles of intrinsically disordered proteins (IDPs) in biology were not recognized until the late 1990s. Observations accumulated that many functional proteins, particularly those involved in transcription and translation regulation, appeared to be intrinsically unstructured [1]. This prompted Wright and Dyson to publish their seminal review in 1999 [2], calling for a reassessment of the protein structure-function paradigm. Since then, study of IDPs has rapidly evolved into a field of “growing prominence” and “one of the most exciting undertakings of structural biology” as predicted [3,4]. It is now established that IDPs are: 1. highly abundant in biology, with about one-third of eukaryotic proteins predicted to be IDPs [5,6]; 2. frequently involved in crucial areas such as signaling and regulation [7–9]; and 3. extensively associated with human diseases including cancer, diabetes, neurodegenerative diseases and amyloidoses [10,11]. Many IDPs undergo folding transitions upon binding to specific targets [12], even though some IDPs remain unstructured in the bound states [13,14]. The mechanisms of these coupled binding-folding interactions are of significant importance because they underlie the organization of important regulatory networks that inform various aspects of cellular decision-making, cellular fate, and cellular signaling. Diseases and disorders can result from IDP mis-folding as well as mis-signaling and mis-regulation [11]. While the prevalence of IDPs as functional proteins was not fully recognized until the 1990s as stated above, one reviewer pointed out several pieces of work that were early hargingers. To illustrate this, three examples have been chosen here from a much larger collection. As early as 1953, optical rotation suggested that the milk protein casein is similar to globular proteins unfolded by 5 M guanidinium hydrochloride [15]. By the mid-1960s, optical rotation was used to develop a conformational classification of proteins, which included proteins that were characterized as disordered [16]. By the early 1970s, studies on fibrinogen strongly indicated that this protein contained large regions that lack structure [17], and more recently, this early history was reviewed in a very interesting fashion [18].

For signaling and regulation, the disordered nature of IDPs is believed to offer several unique advantages [2,3], including high specificity/low affinity binding, inducibility by posttranslational modifications, and structural plasticity for binding multiple targets. The last property appears to be a hallmark of IDPs that allows one-to-many and many-to-one signaling [19,20]. Intrinsic thermo-instability can also offer a robust mechanism for allosteric coupling [21]. However, the molecular mechanism of the coupled binding and folding interaction of IDPs has remained largely elusive except in a very small number of cases [22–24]. In particular, while it is recognized that residual structures often persist in unbound IDPs [7], their functional implications are under much debate [25]. Clearly, the extent of residual structure modulates the entropic cost of folding, and thus affects the binding affinity. It has been further proposed that preformed structural elements might serve as initial contact points and facilitate IDP folding on the substrate surface [26,27]. On the other hand, increasing the amount of local structures in the unbound state actually reduces the binding rate for several IDPs [28,29], emphasizing the importance of intrinsic flexibility in facile recognition of IDPs.

Important progresses have been made over the last ten years or so in prediction, identification and general characterization of IDPs [30–34]. In particular, sequence analysis can be quite reliable applied to predict disordered protein segments with averaged sensitivity and specificity scores greater than 0.8 [35]. The Database of Protein Disorder (DisProt, version 5.3, 09/21/2010) currently contains 1,284 experimentally verified disordered regions within 594 proteins, and over forty atomistic structures of IDP complexes are now available [36]. One of the key challenges in IDP research at present is to understand mechanistically how intrinsic flexibility is exploited together with nascent structures and other biophysical signatures of IDPs for biological functions such as efficient and versatile binding. Such understanding is necessary for deciphering the elaborate physiological control of IDP function and how such control might fail in human diseases. This review will discuss the key challenges in experimental characterization of unbound IDPs, how physics-based *de novo* simulations might help to provide important missing details of IDP structure and interaction, and important lessons that have been derived from a limited number of recent *de novo* simulations of regulatory IDPs. We note that a significant amount of computational work exists on studies of disordered proteins involved in protein aggregation and amyloidogenesis [37]. This review focuses on signaling and regulatory IDPs that often undergo coupled binding and folding during function. Studies of amyloidogenic proteins will only be discussed in the context of common challenges and opportunities for *de novo* simulations.

## 2. Challenges in Detailed Experimental Characterization of IDPs

Structural information of the bound state alone is not sufficient to establish biologically relevant regulatory mechanisms. For IDPs, the nature of the unbound state holds important clues to function. Due to the heterogeneous and dynamical nature, detailed characterization of unbound IDPs has proven to be a principal challenge. Consequently, a lack of understanding of the nature of residual structures in unbound IDPs hinders further clarification of their functional roles. Among a wide range of biophysical techniques available for characterizing disordered protein states [34], biomolecular NMR is probably the most comprehensive [38]. Many observables can be measured for multiple sites throughout the protein to infer (transient) organizations at the secondary and tertiary levels, including: chemical shift, coupling constant, nuclear Overhauser effect (NOE), residual dipolar coupling (RDC), paramagnetic resonance enhancement (PRE), and spin relaxation. Chemical shifts, coupling constants and NOEs can be used to determine secondary structure propensities semi-quantitatively at the residue level [39–42]. PRE and RDC are powerful tools for uncovering the existence of transient tertiary organizations [43–45]. They have been applied to derive important insights on many disordered protein states (see recent reviews [7,38,46,47]). However, a quantitative, structural interpretation of these experimental observables measured on unbound IDPs is generally not feasible. A key issue is that only ensemble-averaged properties can be measured in general and they must be represented as averaged quantities of a heterogeneous structure ensemble. Coupled with typical scarcity of data, the structural calculation is severely underdetermined and a unique structure ensemble cannot be determined solely from the experimental restraints. This critical limitation has not been fully appreciated in the literature. Certain intrinsic properties of RDC and PRE further complicate the structural interpretation. For example, PRE is extremely sensitive to the electron-nucleus distance with *r*^−6^ dependence [48]. While such sensitivity allows detection of weakly populated transient contacts, it also renders PRE largely insensitive to the majority of accessible conformations. Using theoretical PRE data sets derived from simulated disordered protein states, we recently demonstrated that ensemble structural calculation protocols with PRE distance restraints [49,50] could generate misleading ensembles that reflect little on the true underlying protein state [51]. Direct incorporation of RDC in disordered ensemble calculation has not been feasible because of the intricate relationship between observed data and individual ensemble structure, even though strategies exist for globular proteins [52–54]. Thus, RDC has been mainly used for validation of various (coil) models of unfolded states [55,56] or as filters to select conformations from pre-generated structural pools [57,58]. Importantly, this general difficulty in structural interpretation arises mainly from the fundamental limitations of ensemble-averaged properties measured on disordered protein states rather than technical ones. There is simply not sufficient constraint for independent identification of representative ensembles from experimental restraints alone. A few exceptions may include cases where additional information on the accessible conformational substates is available or can be assumed [59,60], or where unusually large amount of experimental data were measured [61].

## 3. Opportunities and Challenges for *de Novo* Simulations

The substantial challenge in detailed experimental characterization of IDPs presents a unique and exciting opportunity for molecular modeling to make critical contributions. In particular, atomistic simulations using physics-based empirical molecular mechanics force fields [62,63] arguably provides the ultimate level of detail necessary for understanding disordered protein states. The experimental data can be the used as independent validation of simulated ensembles instead of as restraints. Such a strategy avoids the under-determined structural calculation problem that tends to generate a false sense of excellent agreement with experiment. At the same time, the dynamic and unstructured nature of IDPs also presents substantial new challenges, pushing the limit on both force field accuracy and conformational sampling capability. Traditional explicit inclusion of water molecules arguably provides the most realistic description of solvent, but also significantly increases the system size (∼10–fold). This can lead to prohibitive computational cost if one wants to sufficiently sample the broad manifold of functionally relevant states of IDPs. Moreover, mainly optimized for folded native states, current explicit solvent protein force fields are known to suffer from systematic biases in describing peptide conformational equilibria, such as tendency to over-stabilize helices [64,65] and peptide-peptide interactions [66]. These existing force field limitations need to be carefully considered in simulations of IDPs. Thanks to development of powerful computational hardware and advanced sampling techniques, important advances are being made recently in optimization of explicit solvent protein force fields [67–69]. Nonetheless, correcting the systematic biases in a consistent and transferable fashion has been difficult, and the computational cost is one of the main obstacles.

Alternatively, implicit solvent has emerged as a powerful approach for atomistic simulation of protein conformational equlibria that provides a balance between description of the essential physics and computational feasibility [70]. The principle and practice of implicit solvent is well documented [70,71]. The basic idea is to capture the mean influence of water on the solute via direct estimation of the solvation free energy. As such, only the solute is represented atomistically, and the system size is reduced ∼10-fold. Important advances have been made recently to greatly improve the efficiency and achievable accuracy of implicit solvent, particularly with the generalized Born (GB) approximation. For example, protein simulations in the GBSW implicit solvent is ∼30 times faster than equivalent ones in explicit water [72]. Importantly, a substantial gain in efficiency allows careful optimization of implicit solvent protein force fields to suppress certain systematic biases that have plagued explicit solvent ones [62]. The key is to capture the delicate balance of competing solvation and intramolecular interactions on the peptide and protein level. We recently re-balanced the GBSW protein force field based on pair-wise interactions of side chain analogs and conformational properties of model peptides [73]. The optimized force field does not only recapitulate the experimental structures *and* stabilities of several helical peptides and a series of β-hairpins with a wide range of stability, but also folds hairpin trpzip2 and mini-protein Trp-cage [73]. The same force field has also been successfully applied to pKa prediction [74], pH-dependent protein folding [75–77], structure refinement [78], and recently to simulation of regulatory IDPs [79,80]. Similar optimization efforts have also led to substantial improvement in other GB models [81,82]. An ABSINTH implicit solvent has also been developed and optimized specifically for IDPs [83].

It is important to recognize that inherent and methodological drawbacks do exist in implicit solvent [70]. They need to be carefully considered in the interpretation of implicit solvent simulations. This is particularly important considering that validation based on ensemble-averaged properties is not conclusive because of the same reasons detailed above for difficulty in structural calculations. Implicit solvent will not properly describe short-range effects where the detailed interplay of a few non-bulk-like water molecules is important. With a lack of solvent granularity, continuum models do not capture all the fine structures in potentials of mean force (PMFs) of interactions [73,84], and the conformational diffusion kinetics is altered. The temperature dependence cannot be described accurately in general either. Besides these intrinsic limitations, implicit solvent might be further limited by the specific methodology for calculating the solvation free energy as well as the physical parameters of the solvation model and underlying protein force fields. In particular, the surface-area (SA)-based treatment of nonpolar solvation appears to be a key methodological limitation in the current GB implicit solvent [85,86]. Nevertheless, a substantial reduction in the computational cost without compromising the essential physics is an important advantage of implicit solvent. By taking care in interpretation and validation of simulations, one can expect that *de novo* simulations to provide reliable details on the structure and interaction of IDPs.

Even with the dramatic reduction in system size using implicit solvent, it is challenging to sufficiently sample biologically accessible, functionally relevant conformational space of IDPs. Conventional constant temperature molecular dynamics (MD) is generally insufficient for achieving convergence in simulated structural ensembles of IDPs. The difficulty arises not only because of the large and complex conformational space of proteins, but also due to significant energy barriers that might separate different conformational subspaces. It is necessary to exploit various advanced techniques to enhance sampling [87,88]. One particularly simple yet effective technique is replica exchange (REX) [89]. The basic idea is to simulate multiple independent replicas of the system at different temperatures, typically distributed exponentially between the temperature of interest and a maximum temperature. Periodically, replicas attempt to exchange simulation temperatures according to a Metropolis criterion that preserves the detailed balance and ensures proper canonical ensembles at all temperatures. The resulting random walk in the temperature space helps the system to avoid being kinetically trapped in states of local energy minima. The required number of replicas increases with the system size N (as a function of its squared root [89]). With ∼10-fold smaller system size, implicit solvent is thus particularly suitable for REX. Recent theoretical considerations [90–92] and actual simulations of small peptides [93–96] generally confirm that REX can enhance protein conformational sampling as long as the activation enthalpies (of conformational transitions) are positive. In particular, it is important to specify a maximum REX temperature slightly above where the folding rate maximizes [91,92]. The key to obtain well-converged structural ensembles of IDPs is efficient sampling of transitions between conformational substates. However, the nature of these substates and the energy barriers of their inter-conversion are not known. Therefore, the efficacy of REX for IDPs is not very obvious, nor is the optimal choice of key REX-MD parameters such as the number of replicas, range and distribution of simulation temperatures and exchange attempt frequency. On the other hand, several recent experimental studies suggest that the average roughness of protein energy surface likely exceeds 5 RT [97–99], which can lead to a strong temperature dependence of conformation diffusion [100]. The roughness consideration argues strongly for the general ability of REX to enhance sampling of protein conformations including those of IDPs.

Another particularly attractive approach to overcome the sampling bottleneck is to combine large numbers of equilibrium and/or generalized ensemble simulations (e.g., on distributed computing platforms [101]) using network methods like the Markov State Models (MSMs) [102–104]. Even though it is yet to be applied to IDPs, this strategy has provided unprecedented detail on energy landscapes of several proteins under both stable and unstable conditions [105–107]. It is important to emphasize that sampling efficiency also strongly depends on the force field quality. In particular, modern protein force fields, with explicit or implicit solvent, tend to overestimate the strength of protein-protein interactions [66]. Consequently, non-specific collapsed protein states are often over-stabilized, which severely hinders conformational sampling. When the goal is not to generate proper thermodynamic ensembles at physiological temperatures, temperature-induced unfolding/unbinding simulations can be used to infer on the mechanism of coupled binding and folding of IDPs given the complex structures. This strategy has been quite successful in studies of protein folding [108,109], and has already been applied to understand IDP interactions [110,111]. An interesting advantage of high-temperature simulations is that they are less sensitive to imperfections in the force fields, besides reduced computational cost. A key concern is that the transition states or the most probable transition paths might depend on the temperature. However, IDP complexes tend to be less stable than globular ones, and the temperature required for unbinding simulations will likely be moderate.

## 4. Key Lessons from Recent *de Novo* Simulations of Regulatory IDPs

### 4.1. Unbound IDPs: Nascent Structures and Dependence on Post-Translational Modifications

As discussed above, the nature of the unbound state of an IDP holds important clues to how it might interact with specific targets. Abundant experimental evidence exists to suggest that various levels of residual structures persist in unbound IDPs. Such examples include several domains of transcription factor CREB [7], activation and regulatory domains of tumor suppressor p53 [112–114], the steroid receptor coactivator ACTR [115], the synuclein family [116,117], and cyclin-dependent kinase (Cdk) regulators p21 and p27 [118,119]. A key goal of *de novo* simulations of IDPs has thus been to provide molecular detail of such residual structures in unbound IDPs. In the following, we summarize key results from several recent *de novo* simulations of free regulatory IDPs where the goal is to understand coupled binding and folding. We note that physics-based simulations have also provided important insights into the intrinsic conformational properties of IDPs and how they might depend on certain physiochemical properties [120,121]. In particular, IDPs are known to have low sequence complexity with enriched charges. Several recent studies have shown that the structure of IDPs is correlated with the charge content [121,122]. It has been further suggested charges on IDPs can directly affect function, in both protein-DNA [123] and protein-protein interactions [80].

**Figure 1.**
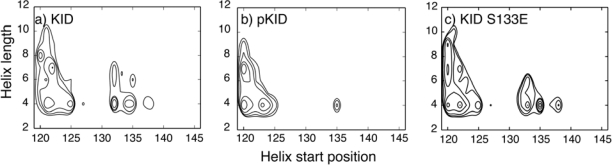
2D probability distributions of helix starting positions and lengths in the unbound structure ensembles of (a) KID, (b) pKID and (c) KID S133E at 302 K calculated from REX-MD folding simulations in GBSW implicit solvent [79]. Contours are drawn at 0.002, 0.005, 0.01, 0.02, 0.04, and 0.06 levels.

The kinase-inducible domain (KID) of CREB is one of the most extensively studied IDPs. In cell, CREB regulates transcription in response to cAMP signaling partially by binding to the coactivator CBP (CREB binding protein) [124]. This recognition requires phosphorylation of Ser133 of KID (pKID) and involves direct interaction with the KIX domain of CBP [125,126]. When in complex with KIX, pKID adopts a helix-linker-helix structure that involves residues 119 to 146 [127]. However, free KID and pKID lack stable tertiary structures in solution. NMR chemical shift analysis has estimated that helix αA (residues 120–129) is about 50–60% folded and helix αB (residues 134–144) is only 10–15% formed [128]. Furthermore, secondary chemical shift analysis does not reveal significant differences in mean residue helicities upon phosphorylation [128]. Accordingly, the principal role of pSer133 has been attributed to mediating intermolecular interactions. Slot *et al*. have investigated the intrinsic structural properties of the unbound KID and pKID using explicit solvent MD simulations [129]. It was argued that phosphorylation could reduce the loop flexibility connecting two helices in the bound structure and induce a transient structural element that resembles native-like conformation. However, these simulations were conducted by using a simulated annealing protocol in explicit solvent. The total simulation length is only 2.0 nanoseconds (ns) with the last 1.0 ns as the production stage at 300 K. This is too short to sufficiently sample relevant conformations in the disordered state, as reflected in the narrow backbone φ/ψ distributions reported [129]. Persistence of native-like loop conformation in pKID could be an artifact of limited sampling initiated from the folded structure. This concern substantially weakens the reliability of the proposed effects of phosphorylation. Well-converged structural ensembles of KID and pKID were later calculated using 200-ns REX-MD simulations in the optimized GBSW force field [73]. The simulated ensembles are mainly validated by comparing the mean residue helicities with the NMR results. Further structural analyses show that both KID and pKID are compact and mainly occupy a small number of helical substates (see Figure 1). Interestingly, even though in agreement with NMR pSer133 only leads to marginal helicity changes on the ensemble level, the underlying conformational substates differ significantly. In particular, pSer133 appears to restrict the accessible conformational space of the loop connecting two helical segments and thus can reduce the entropic cost of KID folding upon binding to KIX. This entropic contribution supplements the salt-bridge interactions between KID pSer133 and KIX Lys662 and Tyr658. Replacing pSer133 with a Glu residue fails to induce similar structural changes (see Figure 1c). This explains why KID S133E does not bind to KIX nearly as strong as pKID [130], even though Glu can interact with similar strength with Lys or Tyr [131]. However, in contrast to Slot *et al*.’s simulations, pS133 does not appear to induce native-like loop conformations. The expanded role of phosphorylation in regulating the KID/KIX recognition was not recognized in the NMR studies due to lack of experimentally detectable conformational changes on the ensemble level. This highlights the importance of combining simulation and experiment for detailed characterization of unbound IDPs. The ability of well-converged *de novo* simulations to “predict” important features of the disordered states of a 28-residue IDP is nontrivial and represents an encouraging progress towards accurate simulation of IDP conformational equilibria.

REX-MD simulations in implicit solvent have also been used to compute the structure ensemble of unbound p53 extreme C-terminus [80]. The results demonstrate that the free p53 peptide samples several distinct conformations, which, importantly, correspond to those experimentally observed when bound to different partners [19]. Presence of folded-like nascent structures has also been suggested in integrated computational and experimental studies of several other regulatory IDPs. Kriwacki and coworkers combined NMR data with a 100-ns explicit solvent simulation and suggested that sub-domains within free p27 kinase inhibitory domain exhibit partially folded nascent structures that resemble the bound conformation observed in the ternary complex of p27/Cdk2/cyclin A [118]. Yoon *et al*. characterized the residual structural elements of the C-terminal segment of p21, also using a combination of NMR and MD simulations, and identified helical and extended conformations that resemble those in complex with either calmodulin or proliferating cell nuclear antigen (PCNA) [119]. However, the role of nascent structures within these free IDPs in binding is not obvious, even though one might be tempted to speculate that such preformed structures might facilitate binding by providing initial binding sites (i.e., conformational selection-like mechanisms) [26,27].

### 4.2. Mechanism of Coupled Binding and Folding: Intrinsic Flexibility *vs.* Preformed Structures

Compared to studies of unbound IDPs, there are even fewer *de novo* computational analyses of the molecular mechanisms of coupled binding and folding of IDPs. This can be primarily attributed to much greater computational cost. Rigorous thermodynamic and/or kinetic characterization of coupled binding and folding of IDPs is generally not feasible. In a pioneering study, Verkhivker *et al*. investigated the mechanism of p27 binding to Cdk2/cyclin A using high-temperature unfolding and unbinding simulations with a simplified all-atom protein energy function with physics-motivated terms [132]. The calculated transition state ensemble (TSE) suggests an induced folding-like mechanism, where molecular recognition is initiated by formation of nonspecific encounter complexes with unstructured p27. This finding supports the “fly-casting” mechanism proposed by Wolynes and coworkers [133,134], which argues that nonspecific binding of unstructured conformations confers a kinetic advantage for binding. Another key lesson obtained from this study is that intermolecular interactions appear to dedicate the folding mechanism of p27 and overwhelm local folding preferences in unbound p27. This conclusion might prove to be applicable to other regulatory IDPs. In particular, structural plasticity for adopting distinctive folded states is believed to be a hallmark of regulatory IDPs. Therefore, the binding partner ultimately determines the IDP topology in the bound state, *i.e.*, intermolecular interactions do overwhelm local folding preferences of IDPs prior to binding. Indeed, induced-folding was also observed in a recent high-temperature simulation of pKID/KIX [135].

**Figure 2.**
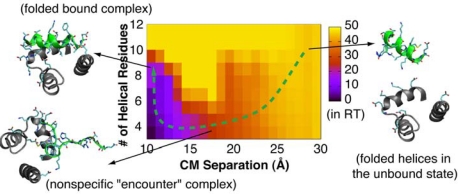
2D free energy surface of the folding and binding of p53 to S100B(ββ). Folding is described by the number of helical residues, and the binding by the center-of-mass (CM) separation [80]. Representative structures are shown for regions that correspond to the unbound, nonspecific contact, and bound states. Charged residues on p53 and near its binding site on S100B(ββ) are shown in sticks

The only attempt to rigorously calculate the binding and folding free energy surfaces of IDPs reported so far is on the p53/S100B(ββ) interaction [80]. A protocol that combines REX and umbrella sampling was used, and several approximations were necessary to further reduce the computational cost even with implicit solvent. In contrast to the speculation of conformational selection based on the presence of substantial helical content in the unbound state, the calculated energy surfaces reveal that the p53 extreme C-terminus initially binds to S100B(ββ) in an unfolded conformation and then quickly folds - an example of induced folding (e.g., see the green path in Figure 2). The residual structure in the unbound state thus mainly modulates the binding affinity, while the intrinsic flexibility is critical for the binding rate. This study illustrates that the presence of folded-like conformations in unbound IDPs is not sufficient evidence to establish conformational selection. It is important to directly examine the thermodynamics and/or kinetics of coupled binding and folding for mechanistic characterization. This study also reveals important remaining limitations in the current implicit solvent force field, which does not only substantially over-estimate the interaction strength but also fails to predict the experimental structure as the global free energy minimum.

## 5. Conclusions

IDPs are an important class of functional proteins, with high abundance and fundamental roles in biology and broad association with human diseases. Important progresses have been made in the prediction, identification and high-level characterization of disordered protein segments. Nevertheless, much needs to be learned about the nature of the unbound state and the molecular mechanism of coupled binding and folding. Detailed experimental characterization of IDPs is challenging due to the heterogeneous and dynamic nature. IDP thus represents an exciting opportunity for physics-based modeling to make critical contributions. At the same time, simulation of IDP presents substantial new challenges that push the limit on both protein force field accuracy and conformational sampling capability. New modeling strategies need to be explored to address these challenges. Implicit solvent, coupled with advanced sampling techniques, provides a particularly suitable means for *de novo* characterization of IDP structure and interaction with a necessary balance between accuracy and efficiency. Only a small number of *de novo* simulations have been reported on regulatory IDPs so far, reflecting the existence of substantial challenges in the current simulation methodologies. Nonetheless, important insights have been obtained on the nascent structures and how they might contribute to molecular recognition of IDPs. With the sampling limitation alleviated by ever-increasing computing power and sophisticated algorithms that exploit abundant computing resources, the critical bottleneck for physics-based simulations of IDPs is in the achievable level accuracy of current protein force fields. This underpins intensive ongoing efforts in improving the ability of explicit and implicit solvent protein force fields to describe protein conformational equilibria. It is important to diligently validate *de novo* simulations using available experimental data and proper (positive and negative) controls. We anticipate an integrated computational and experimental strategy to address specific questions on the structure and interaction of biologically important IDPs, and are excited by the unique role that physics-based simulations will be expected (and required) to play.
